# Editorial: Nano-(bio)sensors for on-site monitoring: Advancing diagnostics through technological intervention

**DOI:** 10.3389/fbioe.2024.1475130

**Published:** 2024-08-30

**Authors:** Anupriya Baranwal, Sharmili Roy, Ashutosh Kumar

**Affiliations:** ^1^ Department of Applied Chemistry and Environmental Science, RMIT University, Melbourne, VIC, Australia; ^2^ Department of Medicine, School of Medicine, Stanford University, Stanford, CA, United States; ^3^ Department of Electrical Engineering, University of Notre Dame, Notre Dame, IN, United States

**Keywords:** biosensors, electrochemcial sensor, nanobiosensors, point - of - care diagnostics, nanomaterials, healthcare diagnostics

Nano-(bio)sensors, utilizing the distinctive characteristics of nanomaterials, have emerged as revolutionary tools for on-site monitoring, be it in healthcare, food safety, or environmental tracking (Kulkarni et al., 2022; Tripathi and Bonilla-Cruz, 2023; Rasheed et al., 2024). These sensors combine (bio)molecular recognition elements (MREs) and nanomaterials to enable precise monitoring of analytes with exceptional sensitivity and specificity (Kumar et al., 2018; Mahato et al., 2018). Advances in nanotechnology, material science, and microfabrication have made it possible to develop miniaturized (bio)sensors more easily now than ever (Kumar et al., 2019a; 2019b; Roy et al., 2019). Anticipating progress in this field, this Research Topic has been curated to showcase the most recent developments and viewpoints on nano-(bio)sensors and their applications. This Research Topic consists of three reviews and four original research articles demonstrating the transformative impact of nano-(bio)sensor technologies in enhancing diagnostic accuracy, speed, and accessibility, paving the way for advanced diagnostic interventions and precision medicine.

Point-of-care (POC) tests are diagnostic tools designed for use at or near the site of patient care, providing rapid results that enable immediate clinical decisions. POCTs can be essential in early clinical diagnosis and personalized patient care, significantly reducing disease outbreaks and improving patient survival rates (Zarei, 2017; Baranwal et al., 2024). The importance of POCT was highlighted during the COVID-19 pandemic with the widespread use of rapid antigen test (RAT) kits, which provided quick and accessible testing, crucial for controlling the virus’s spread. While the incorporation of gold nanoparticles in commercial lateral flow devices and immunoassays is widely exploited for the ease of bioconjugation and improving sensitivity, no other nanomaterial has yet been able to see a similar success. In this direction, the use of magnetic nanoparticles (MNPs) in (bio)sensor platforms is gaining tremendous attention since these nanoparticles can aid in target analyte capture, isolation, and enrichment, thereby enhancing sensor performance. Owing to their high sensitivity and swift detection capability, these (bio)sensors may soon find a niche in infectious disease diagnoses and chronic disease management. Wang et al., in their review, evaluated the current POCT landscape, highlighting its increasing importance in clinical practice. They discussed the integration of MNPs into various diagnostic platforms and assessed their potential impacts on platform performance.

Precision medicine is an approach that customizes healthcare by tailoring treatments to individual patients based on their genetics, environment, and lifestyle (Evans et al., 2024). Unlike traditional medicine, which often relies on generalized treatments, precision medicine aims to move beyond a one-size-fits-all approach to provide more effective and targeted care. The intersection of advanced technology and healthcare has launched a transformative era aimed at precision medicine. Leading this charge are wearable and implantable biosensors, as they enable real-time, personalized health monitoring, helping in the early detection of issues, offering tailored healthcare solutions, and enhancing patient outcomes (Li et al., 2021; Lin et al., 2021). Continuous glucose monitors and wearable ECG monitors are prime examples of gadgets that provide real-time health data to manage chronic illnesses. Ghazizadeh et al., explored the recent advancements in the field of wearable and implantable biosensors and highlighted their potential to revolutionize precision medicine. They discussed existing and potential challenges associated with these sensors and provided insights on overcoming them in the future.

In recent years, innovative designs and nanomaterial integration have considerably advanced ion-selective optodes (ISOs). Barhoum et al., in their review, investigated contemporary ISOs with an emphasis on developing strategies to improve real-time monitoring, selectivity, and sensitivity. The article addressed conventional membrane-based optodes and examined the most recent research, design principles, and critical components, including ionophores, indicator pigments, polymer membranes, and nanomaterials. Nanomaterials, including quantum dots and rare earth elements, were prioritized due to their potential to improve ISOs. The review also emphasized the development of innovative designs, such as disposable paper-based optodes, smartphone-based optodes, and wearable optodes.

The research work by Xiao et al., demonstrated a loop-mediated isothermal amplification test to diagnose *Moraxella catarrhalis* infection quickly and accurately. This progress is vital for clinical diagnostics in respiratory diseases, as prompt and precise identification can lead to quick intervention, greatly influencing patient outcomes. The ability to detect multiple analytes simultaneously or sequentially can be a notable advancement, as it allows for comprehensive diagnoses using a solitary test, thereby reducing time and resources while enhancing diagnostic precision. In this direction, Yang et al., developed a multiplex testing system that can detect three respiratory pathogens simultaneously: SARS-CoV-2, influenza A, and influenza B. The test consisted of three main components: a sample extraction and enrichment module, an ultra-fast PCR module, and a smartphone-based data processing module. The sample extraction and enrichment module helped improve the system’s sensitivity and specificity by extracting nucleic acids efficiently and reducing impurities and preparation time, all without needing a large sample volume. The real-time fluorescent PCR allowed rapid nucleic acid amplification and qualitative detection of pathogen RNAs through changes in fluorescence signals. Finally, the smartphone processed and analyzed the signals quantitatively and detected all three pathogens simultaneously.

Apart from pathogens, the detection of clinically relevant biomarkers with high sensitivity and precision is also desired. Dopamine is a neurotransmitter that plays a crucial role in regulating various body functions, including emotions, memory, and movement. Alterations in dopamine levels can cause neurological diseases (e.g., Parkinson’s and Alzheimer’s) and affect mental health (Baranwal and Chandra, 2018). Nishan et al., developed a simple, rapid nanozyme-based biosensor for colorimetric detection of dopamine. Cobalt-doped hydroxyapatite was exploited as a peroxidase-mimic to oxidize TMB substrate into blue color product by using H_2_O_2_. The sensor worked on an on/off strategy, where the absence of dopamine was confirmed by a blue color (on-state); however, its presence was confirmed by the disappearance of color (off-state). In another original work, Zaw et al., fabricated an electrochemical biosensor for monitoring glycated albumin (GA)– an indicator of glycemic control in diabetic patients. Polydopamine nanoparticles (PDA NPs) conjugated with GA-specific aptamer were used to coat the electrode surface and generate a GA concentration-dependent response with high sensitivity. The molecular dynamics simulations were used to gain insights into how PDA NPs interacted with aptamer during conjugation in order to improve the sensor design.

Nano-(bio)sensors represent a highly impactful technological advancement in the diagnostic sector, offering unparalleled sensitivity, specificity, and adaptability for real-time monitoring at the desired location. Ongoing advancements in material design and sensor fabrication strategies, including, but not limited to, screen-printing, inkjet printing, gravure printing, and lift-off lithography, have made these biosensors indispensable tools in healthcare, food safety, and environmental monitoring. Despite these advancements, several scientific and engineering challenges persist, which can be broadly divided into two categories: those associated with nanomaterials and MREs and those associated with sensor platforms. Challenges related to nanomaterials include uniformity, batch-to-batch variation, stability, scalability, and biocompatibility. On the contrary, challenges associated with sensor platforms include cost, accuracy, reliability, reproducibility, turn-around time, user-friendliness, miniaturization, and shelf-life. Fabrication of integrated devices capable of meeting all the requirements of direct interaction with biological systems is still an ongoing challenge. Additionally, the lack of standardized procedures and regulatory frameworks further hinders their widespread adoption. In order to ensure the successful deployment of nano-(bio)sensors for commercial application, the aforementioned challenges must be overcome. Clear guidelines must be established for developing, testing, and validating (bio)sensor platforms to warrant their safety and reliability. Automation of sample handling and pretreatment by integrating (bio)sensors with microfluidic technology could achieve rapid and reliable results, thereby enhancing biosensor precision. Integrating artificial intelligence and machine learning in (bio)sensor design and data analysis will improve their performance by analyzing complex data patterns and providing predictive insights. Considering the pace of material and sensor technology advancements, nano-(bio)sensors are expected to define the era of diagnostics and monitoring for several years to come.
